# *Adenophora Stricta* Root Extract Protects Lung Injury from Exposure to Particulate Matter 2.5 in Mice

**DOI:** 10.3390/antiox11071376

**Published:** 2022-07-15

**Authors:** Seok-Man Park, Cheol-Jong Jung, Dae-Geon Lee, Beom-Rak Choi, Tae-Hun Ku, Im-Joung La, Il-Je Cho, Sae-Kwang Ku

**Affiliations:** 1 Department of Histology and Anatomy, College of Korean Medicine, Daegu Haany University, Gyeongsan 38610, Korea; smpark@okchundang.co.kr (S.-M.P.); ghost71715@okchundang.co.kr (D.-G.L.); 2 Central Research Center, Okchundang Inc., Daegu 41059, Korea; oc_cjjung@okchundang.co.kr; 3 Research Institute, Nutracore Co., Ltd., Gwanggyo SK Viewlake A-3206, Beobjo-Ro 25, Suwon 16514, Korea; brchoi@nutracore.co.kr; 4 Okchungdang Korean Medicine Clinic, Ulsan 44900, Korea; oc100002@okchundang.co.kr; 5 Atomy R&D Center, Gongju 32511, Korea; imjna@atomyorot.kr

**Keywords:** *Adenophora stricta* root extract (AsE), anti-inflammation, antioxidant, lung injury, particulate matter 2.5 (PM_2.5_)

## Abstract

Chronic exposure of particulate matter of less than 2.5 μm (PM_2.5_) has been considered as one of the major etiologies for various respiratory diseases. *Adenophora stricta* Miq. is a medicinal herb that has been used for treating respiratory diseases in East Asia. The present study investigated the effect of *A. stricta* root extract (AsE) on PM_2.5_-induced lung injury in mice. Oral administration of 100–400 mg/kg AsE for 10 days significantly reduced the PM_2.5_-mediated increase in relative lung weight, but there was no difference in body weight with AsE administration. In addition, AsE dose-dependently decreased congested region of the lung tissue, prevented apoptosis and matrix degradation, and alleviated mucus stasis induced by PM_2.5_. Moreover, cytological analysis of bronchioalveolar lavage fluid revealed that AsE significantly inhibited the infiltration of immune cells into the lungs. Consistently, AsE also decreased expression of proinflammatory cytokines and chemokines in lung tissue. Furthermore, AsE administration blocked reactive oxygen species production and lipid peroxidation through attenuating the PM_2.5_-dependent reduction of antioxidant defense system in the lungs. Therefore, *A. stricta* root would be a promising candidate for protecting lung tissue from air pollution such as PM_2.5_.

## 1. Introduction

Air pollution has been a global issue that adversely affects human health. In particular, long-term exposure to fine particulate matter of less than 2.5 μm of aerodynamic diameter (PM_2.5_) increases the risk of mortality from various diseases, such as respiratory disease, cardiovascular disease, stroke, and diabetes [1,2]. A recent epidemiological study estimates that PM_2.5_ attributes to 4.58 million deaths and 142.52 million disability-adjusted life years globally in 2017 [3]. Because PM_2.5_ can penetrate into the alveoli by bypassing physiological barrier in the respiratory system, the lungs are the primary organs affected by PM_2.5_. In a molecular toxicological aspect, PM_2.5_ contains various toxicants, such as polyaromatic hydrocarbons (PAHs), heavy metals, sulfate, and endotoxin [4]. These toxicants alter vascular permeability and trigger oxidative stress-mediated inflammation [5,6]. Therefore, PM_2.5_ not only increases the incidence of respiratory diseases, but also aggravates them [1,7,8]. Despite global interest, reducing air pollution immediately seems elusive. Therefore, consumption of nutraceuticals to prevent PM_2.5_-mediated pulmonary damage would be an alternative and attractive option. In regards, several medicinal herbs or those-derived phytochemicals have been suggested to possess pulmonary protective effects against PM_2.5_ without serious side effects [5,6,9,10,11].

Adenophorae Radix (“Sasam” in Korean) is the root of *Adenophora stricta* Miq. or its allied plant, *A. triphylla* var. *japonica* (Regel.) Hara [12], and it has traditionally been ingested for removing phlegm caused by chronic bronchitis and old cough, tonifying and nourishing lung’s yin fluid, moistening the lungs, and reducing fever in East Asia including Korea [13,14,15]. In addition, modern pharmacological evidence demonstrates that *A. triphylla* inhibits ovalbumin-induced asthma [16] and ammonium hydroxide-mediated cough [17]. Moreover, *A. triphylla* has potential to reduce obesity in mice fed a high-fat diet [18] and possesses cytotoxic saponins against certain cancer cells [19]. On the contrary, the beneficial effects of *A. stricta* have been rarely studied except for reporting estrogenic activity [20]. Thus, we conducted preliminary screening study to unravel the potential of *A. stricta* as a nutraceutical and found that *A. stricta* root extract was more effective in alleviating PM_2.5_-mediated pulmonary injury than the same dose of *A. triphylla* extract (data not shown). To expand our knowledge for developing *A. stricta* as a potent pulmonary protective nutraceutical, this study aimed to investigate the dose-dependent effect of *A. stricta* root extract (AsE) in a PM_2.5_-instilled pulmonary injury model and explore the relevant mechanisms. Furthermore, the protective effects of AsE were compared to those of dexamethasone (DEXA), a reference drug.

## 2. Materials and Methods

### 2.1. Preparation of A. stricta Root Extract (AsE)

*A. stricta* collected in Bozhou, China was purchased from Wonkwang Co., Ltd. (Youngcheon, Korea) (Lot number, OCD086AtJ2001), and the authenticity was verified by comparing the high-performance liquid chromatography (HPLC) profile with the voucher specimen (Code number, AS2015) deposited in Korea Institute of Oriental Medicine (Daejeon, Korea). Briefly, *A. stricta* root was extracted twice in boiling water. The resulting extract was filtered, concentrated, and spray dried to prepare AsE. The yield of AsE was 42.81%.

### 2.2. Quantification of Vanillic Acid 4-β-D-Glucopyranoside and Total Flavonoids in AsE

Concentration of vanillic acid 4-β-D-glucopyranoside in AsE was analyzed using an Agilent 1100 HPLC system (Agilent Technologies; Palo Alto, CA, USA) with diode array detector (Agilent Technologies; Palo Alto, CA, USA) and CAPCELL PAK ADME-HR (column size, 4.6 × 250 mm; pore size, 5 μm) (OSAKA SODA Co.; Osaka, Japan). Briefly, AsE or vanillic acid 4-β-D-glucopyranoside (Cayman; Ann arbor, MI, USA) was separated in the binary mobile phase comprising 96:4 ratio of 0.05% trifluoroacetic acid and acetonitrile. Flow rate was 1 mL/min, and eluants were monitored at 254 nm of wavelength. The vanillic acid 4-β-D-glucopyranoside content of AsE was quantified by interpolating the peak area showing the same retention time as that of vanillic acid 4-β-D-glucopyranoside in the AsE chromatogram to the standard curve of vanillic acid 4-β-D-glucopyranoside. In addition, flavonoid content was quantified by measuring optical intensity at 415 nm wavelength after AsE was reacted with 0.2% aluminum nitrate and 20 mM potassium acetate for 40 min. Total flavonoids was expressed as mg of baicalein equivalents per gram of AsE, as described previously [21].

### 2.3. Animal Model and Drug Administration

Experimental procedure using laboratory animals was reviewed and approved by the Institutional Animal Care and Use Committee of Daegu Haany University (Approval number, DHU2021-056; Approval date, 23 July 2021). After sixty SPF/VAF Inbred Balb/cAnNCrlOri mice (age, 6 weeks old; gender, male) supplied from OrientBio (Seongnam, Korea) were acclimatized for 7 days, the mice were randomly allocated into six groups (N = 10 per each group): Vehicle, PM_2.5_, PM_2.5_ + DEXA, PM_2.5_ + AsE (400), PM_2.5_ + AsE (200), and PM_2.5_ + AsE (100). All mice were bred under standard conditions with food and water ad libitum. Diesel particulate matter NIST 1650b (Sigma-Aldrich; St. Louise, MO, USA), which is mainly composed of PAHs (e.g., phenanthrene, fluoranthene, pyrene, and those derivatives) and nitro-substituted PAHs (e.g., 1-nitropyrene and nitrophenanthrene derivatives), was suspended in normal saline injection (saline) consisting of 0.9% sodium chloride (DAI HAN Pharm Co.; Ansan, Korea), sonicated for 30 min to avoid agglomeration, and used as PM_2.5_. To induce pulmonary injury, 1 mg/kg of PM_2.5_ was intranasally instilled into the mice on days 0 and 2 (i.e., day 0 = the first day of injecting PM_2.5_). Mice were orally administered with three different doses of AsE (100–400 mg/kg) or DEXA (0.75 mg/kg; Sigma-Aldrich; St. Louise, MO, USA) dissolved in distilled water for 10 consecutive days. On days 0 and 2, the drugs were administered 1 h after PM_2.5_ injection. To maintain the same stress level, the vehicle group was administered saline and distilled water instead of PM_2.5_ and drug, respectively. The concentration of PM_2.5_ and DEXA was chosen according to the previous reports [11,22]. All mice were euthanized 24 h after the last drug administration, and their lungs were collected for subsequent experiments.

### 2.4. Measurement of Body and Lung Weight

All mice were fasted for 12 h on days 0 and 10 to minimize feeding differences, and body weight was measured once a day during the experimental period using an electronic balance (Precisa Instrument; Zürich, Switzerland). In addition, relative lung weight was calculated as a percentage of lung weight to body weight on day 10.

### 2.5. Lung Sampling and Gross Inspection

After left secondary bronchus and right lower secondary bronchus of the isolated lung was ligated using two blue nylon 3-0 NB324 sutures (AILEE; Pusan, Korea), the upper and middle right lobes were used for collecting bronchoalveolar lavage fluid (BALF). In addition, the lower right lobes were used for preparing tissue homogenate. The left lobes were used for extracting total RNA after capturing digital images. Using an automated image analyzer (*i*Solution FL 9.1, IMT *i*-solution Inc.; Bernaby, BC, Canada), the congested region was measured as a percentage of the congested area relative to the left lobe.

### 2.6. Quantitative Polymerase Chain Reaction (qPCR)

Total RNAs were isolated from the left lobes using a Trizol reagent (Invitrogen; Carlsbad, CA, USA). After incubating RNAs with DNase I (Thermo Fisher Scientific; Rockford, IL, USA), cDNA was synthesized from resulting RNAs using a high-capacity cDNA reverse transcription kit (Applied Biosystems; Foster City, CA, USA). Specific regions of mouse B-cell lymphoma 2 (Bcl-2), Bcl-2 associated X (Bax), mucin 5AC (Muc5AC), Muc5B, nuclear factor κB (NF-κB), p38, and β-actin were amplified using StepOnePlus^TM^ real-time PCR system (Applied Biosystems; Foster City, CA, USA). Primer sequences are listed in Table 1, and the relative expression levels of specific genes were quantified by comparing the C_T_ value of β-actin, as previously reported [23].

### 2.7. Enzyme-Linked Immunosorbent Assay (ELISA)

The lower right lobes in phosphate buffered saline were homogenized using Taco^TM^ Prep bead beater (GeneReach Biotechnology; Taichung, Taiwan) and KS-750 ultrasonic cell disruptor (Madell Technology; Ontario, CA, USA), and then centrifuged at 12,500× *g* for 30 min to obtain lung homogenate. Levels of matrix metalloproteinase 9 (MMP-9), MMP-12, acetylcholine, substance P, tumor necrosis factor α (TNF-α), interleukin 6 (IL-6), C-X-C motif chemokine ligand 1 (CXCL-1), and CXCL-2 were measured using commercial ELISA kits (Mybiosource; San Diego, CA, USA), according to the manufacturer’s instructions.

### 2.8. Cytological Analysis

BALF (approximately 0.7 mL) was collected by injecting saline (1 mL) through the tracheal cannula of the ligated right lobe followed by aspiration with a syringe. After staining the cells of BALF with trypan blue (Sigma-Aldrich; St. Louise, MO, USA), the total number of cells was counted using an automated cell counter (Model, Countess C10281; Invitrogen; Carlsbad, CA, USA). In addition, a Cell-DYN3700 hematology analyzer (Abbott Laboratories; Abbott Park, IL, USA) was used to count white blood cells and their specified cells (e.g., lymphocyte, neutrophil, eosinophil, and monocyte).

### 2.9. Measurement of Reactive Oxygen Species (ROS)

ROS production was detected as previously reported with slight modifications [24,25]. Briefly, lung homogenate (10 μL) was incubated with 10 μM of 2′,7′-dichlorofluorescein diacetate (Abcam; Cambridge, MN, USA) at 37 °C for 0.5 h, and the fluorescence intensity emitted from 2′,7′-dichlorofluorescein was monitored at an excitation wavelength of 490 nm and an emission of 520 nm using a VersaMax^TM^ fluorescence reader (Molecular Devices; Sunnyvale, CA, USA).

### 2.10. Measurement of Lipid Peroxidation, Reduced Glutathione Levels, Catalase and Superoxide Dismutase Activity

Levels of lipid peroxidation and antioxidant defense system (e.g., reduced glutathione, superoxide dismutase activity and catalase activity) in lung homogenate were measured according to previously established method [21], and then normalized by protein concentration.

### 2.11. Statistical Analysis

All numerical values were expressed as mean ± standard deviation of ten mice. Means were compared by One-Way ANOVA or Welch’s ANOVA depending on whether equal variances between different groups were assumed. Tukey’s honestly significant difference test and Dunnett’s T3 test were used as post hoc analyses. A statistical test was performed using SPSS Statistics 18 (SPSS Inc.; Chicago, IL, USA), and *p* < 0.05 was considered significant.

## 3. Results

### 3.1. AsE Decreases Pulmonary Congestion Induced by PM_2.5_

**Figure 1:**
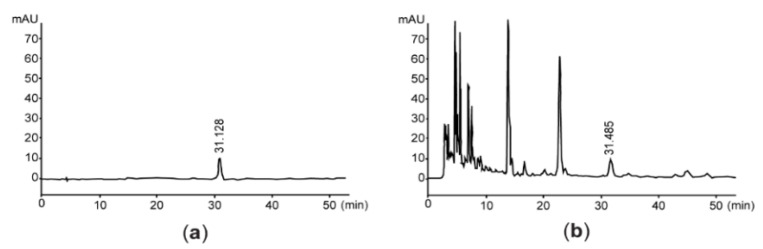
Representative HPLC chromatogram. Eluants are monitored at 254 nm after loading vanillic acid 4-β-D-glucopyranoside (**a**) or AsE (**b**). The number in each chromatogram is the retention time. AsE, *Adenophora stricta* root extract; HPLC, high-performance liquid chromatography.

Since vanillic acid-4-β-D-glucopyranoside is a benzoic acid derivative isolated from the root of *A. stricta* [26], we quantified the level of vanillic acid-4-β-D-glucopyranoside for assessing the quality of AsE. By interpolating the peak area with the same retention time as vanillic acid-4-β-D-glucopyranoside, we found that AsE used in the present study contained 128.58 ± 0.85 μg/g of vanillic acid-4-β-D-glucopyranoside (Figure 1). In addition, flavonoids content was 14.37 ± 0.51 mg baicalein equivalents/g AsE.

**Figure 2:**
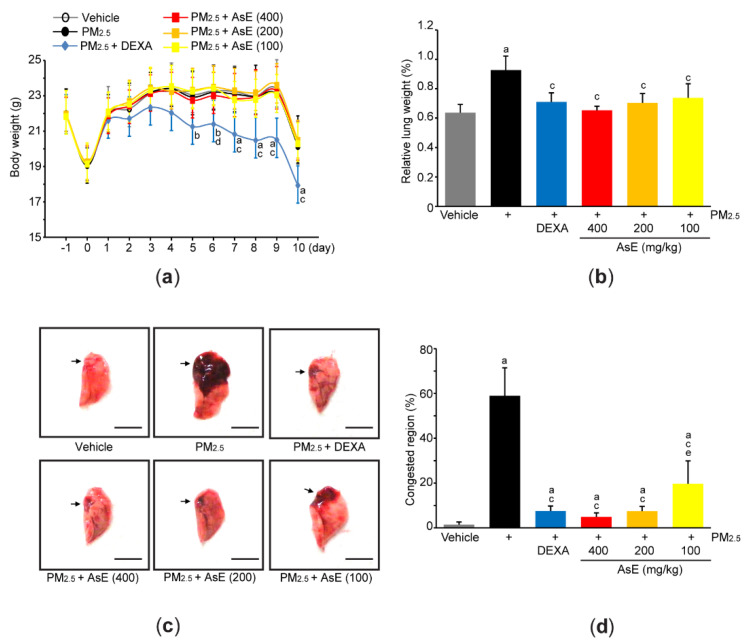
AsE attenuates pulmonary congestion induced by PM_2.5_. (**a**) Changes in body weight were monitored during entire experimental period. (**b**) Relative lung weight was calculated as described in Materials and Methods section. (**c**) Representative image of the left lung lobe. Arrows indicate congested region, and scale bars indicate 6.0 mm. (**d**) Congested region in the left lobe was calculated using an automated image analyzer. ^a^ *p* < 0.01, ^b^ *p* < 0.05 versus vehicle group; ^c^ *p* < 0.01, ^d^ *p* < 0.05 versus PM_2.5_ group; ^e^ *p* < 0.05 versus PM_2.5_ + DEXA group; DEXA, dexamethasone; PM_2.5_, particulate matter 2.5.

Acute pulmonary injury in Balb/c mice was induced by two intranasal injections of PM_2.5_ (1 mg/kg) on days 0 and 2 (i.e., day 0 = the first day of injecting PM_2.5_). To explore pulmonary protective potential of AsE, 100–400 mg/kg AsE were orally administered once daily for 10 days, and 0.75 mg/kg DEXA was used as a reference drug. When comparing the body weight changes among vehicle-, PM_2.5_-, and PM_2.5_ + AsE-administered groups, there were no differences in body weight during the experimental period. On the contrary, significant decrease in body weight was seen from 5 days after the administration of DEXA, as compared to vehicle group (Figure 2a). We and others have already reported that DEXA can reduce body weight [17,27,28], and that this might be due to muscle atrophy [27,28]. Two nasal instillations of PM_2.5_ significantly increased relative lung weight compared to vehicle, while administration of DEXA or three different doses of AsE suppressed the increase in relative lung weight due to PM_2.5_. There was no difference in relative lung weight between the PM_2.5_ + AsE and PM_2.5_ + DEXA groups (Figure 2b). Moreover, gross inspection of lung tissue showed that PM_2.5_ injection significantly increased the area of congested lung. However, DEXA or three different doses of AsE significantly prevented the congestion, and there was no difference in the area of pulmonary congestion between the PM_2.5_ + DEXA, PM_2.5_ + AsE (400 mg/kg), and PM_2.5_ + AsE (200 mg/kg) groups (Figure 2c,d).

### 3.2. AsE Protects Lung Tissue from PM_2.5_-Mediated Injuries

**Figure 3:**
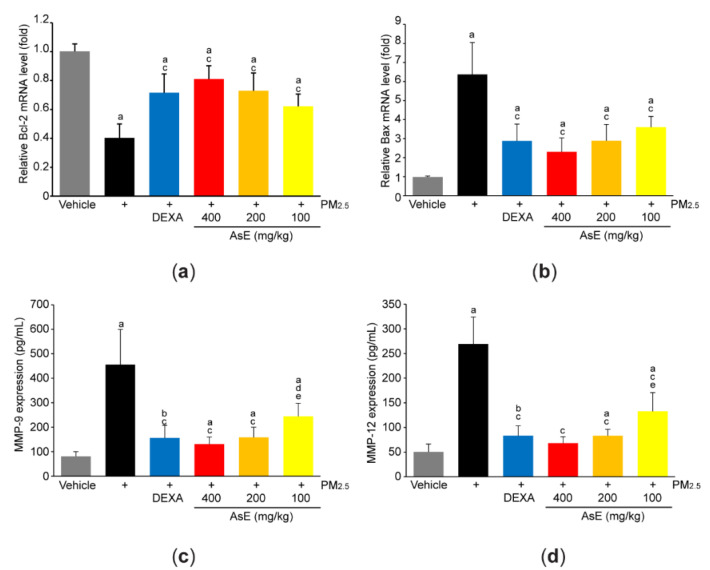
AsE blocks PM_2.5_-induced pulmonary damages. (**a**,**b**) qPCR analyses. Total RNAs isolated from the left lobes were used to quantify expression level of Bcl-2 (**a**) and Bax (**b**). (**c**,**d**) ELISA analyses. Protein homogenate obtained from the lower right lobes were used to quantify expression level of MMP-9 (**c**) and MMP-12 (**d**). ^a^ *p* < 0.01, ^b^ *p* < 0.05 versus vehicle group; ^c^ *p* < 0.01, ^d^ *p* < 0.05 versus PM_2.5_ group; ^e^ *p* < 0.05 versus PM_2.5_ + DEXA group; Bcl-2, B-cell lymphoma 2; Bax, Bcl-2 associated X; ELISA, enzyme-linked immunosorbent assay; MMP, matrix metalloproteinase; qPCR, quantitative polymerase chain reaction.

To explore the role of AsE on PM_2.5_-mediated tissue injuries, mRNA level of representative genes associated with apoptosis was quantified by qPCR analysis. PM_2.5_ significantly reduced Bcl-2 (an antiapoptotic gene) and increased Bax (a proapoptotic gene), suggesting that PM_2.5_ promotes apoptosis in the lungs. However, abnormal expressions of apoptosis-related genes in response to PM_2.5_ were significantly blocked by administrating three different doses of AsE. There was no difference in Bcl-2 and Bax mRNA levels between the PM_2.5_ + AsE and PM_2.5_ + DEXA groups (Figure 3a,b). In addition, PM_2.5_ induced expression of MMP-9 and MMP-12 proteins, which are involved in the loss of elasticity during lung tissue degradation [29]. Administration of three different doses of AsE significantly blocked PM_2.5_-mediated induction of MMP-9 and -12 proteins, and there was no difference in the MMPs reduction between the PM_2.5_ + DEXA, PM_2.5_ + AsE (400 mg/kg), and PM_2.5_ + AsE (200 mg/kg) groups (Figure 3c,d).

Supplementary histopathological results obtained in lung tissue after staining with hematoxylin and eosin showed that PM_2.5_ provoked sarcomatous changes (e.g., reduced alveolar surface area, thickened alveolar septum, and infiltrated inflammatory cells) due to increased PM_2.5_ deposition in lung tissue (Figure S1a). However, PM_2.5_-mediated abnormal changes were significantly blocked by administrating three different doses of AsE (Figure S1a,b). Furthermore, immunohistochemical analyses using an antibody against cleaved caspase-3 (an executive caspase for activating apoptosis) indicated that AsE administration significantly inhibited the increases in the number of cleaved caspase-3 immunoreactive cells by PM_2.5_ in both alveolar and secondary bronchus regions (Figure S1a,c).

### 3.3. AsE Inhibits Mucus Stasis

**Figure 4:**
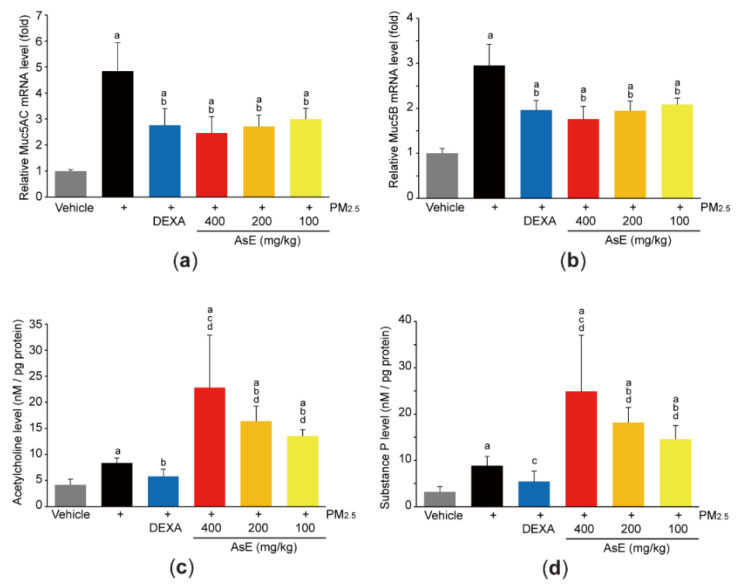
AsE inhibits mucus stasis. (**a**,**b**) Expression level of Muc5AC (**a**) and Muc5B (**b**) in the left lung lobes was quantified by qPCR. (**c**,**d**) Levels of acetylcholine (**c**) and substance P (**d**) in the lower right lobes were determined by ELISA. ^a^ *p* < 0.01 versus vehicle group; ^b^ *p* < 0.01, ^c^ *p* < 0.05 versus PM_2.5_ group; ^d^ *p* < 0.01 versus PM_2.5_ + DEXA group; Muc, mucin.

Mucins are the major O-linked glycoproteins that make up complex and viscoelastic mucus [30], and Muc5AC and Muc5B are polymeric mucins in the airways [31]. To explore whether AsE protects lung tissue by relieving mucus stasis, we firstly quantified mRNA levels of Muc5AC and Muc5B. As expected, PM_2.5_ increased the mRNA levels of Muc5AC and Muc5B. However, administration of three different doses of AsE significantly inhibited PM_2.5_-mediated induction of mucin mRNAs. There was no difference in Muc5AC and Muc5B mRNA levels between the PM_2.5_ + AsE and PM_2.5_ + DEXA groups (Figure 4a,b). In addition, PM_2.5_ instillation slightly, but significantly, increased the levels of acetylcholine and substance P, which are neurotransmitters associated with facilitating mucus secretion [32,33]. Interestingly, AsE administration significantly potentiated PM_2.5_-induced increases in acetylcholine and substance P levels in a dose-dependent manner, while DEXA decreased both neurotransmitter levels (Figure 4c,d). Similarly, our supplementary results after staining lung tissue with periodic acid–Schiff (PAS) showed that AsE further escalated the number of PAS-positive mucus producing cells in the secondary bronchus region compared with the PM_2.5_ group (Figure S2a,b).

### 3.4. AsE Reduces PM_2.5_-Mediated Inflammation in the Lungs

**Figure 5:**
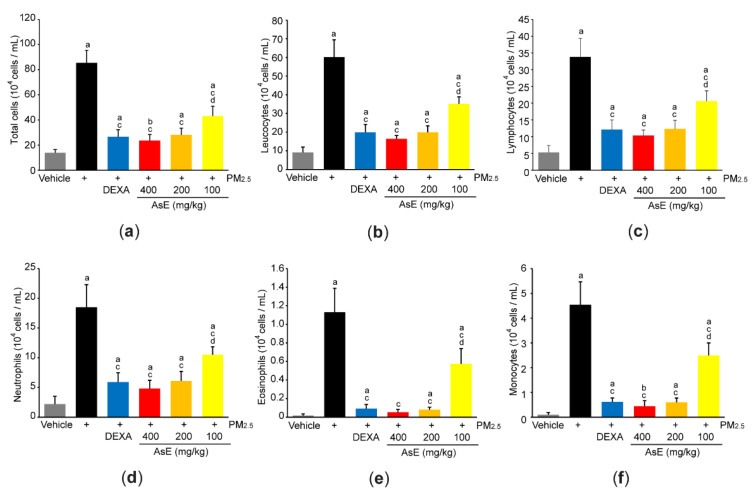
AsE reduces inflammatory cell infiltration in the lungs. (**a**–**f**) Cytological analysis. Number of total cells (**a**), leukocytes (**b**), lymphocytes (**c**), neutrophils (**d**), eosinophils (**e**), and monocytes (**f**) were counted using BALFs obtained from the upper and middle right lobes. ^a^ *p* < 0.01, ^b^ *p* < 0.05 versus vehicle group; ^c^ *p* < 0.01 versus PM_2.5_ group; ^d^ *p* < 0.01 versus PM_2.5_ + DEXA group; BALF, bronchoalveolar lavage fluid.

In parallel with histopathological result that AsE inhibited the number of inflammatory cells in the lung parenchyma (Figure S1b-lower panel), cytological analysis using the BALF revealed that three different doses of AsE significantly reduced increases in total cell, leukocyte, lymphocyte, neutrophil, eosinophil, and monocyte counts in response to PM_2.5_. There was no difference in cell numbers between the PM_2.5_ + DEXA, PM_2.5_ + AsE (400 mg/kg), and PM_2.5_ + AsE (200 mg/kg) groups (Figure 5a–f).

**Figure 6:**
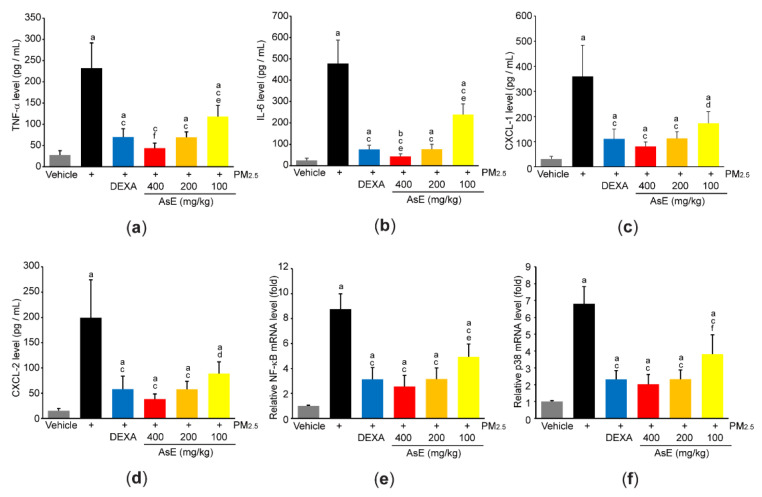
AsE decreases the production of inflammatory mediators in the lungs. (**a**–**d**) Protein levels of TNF-α (**a**), IL-6 (**b**), CXCL-1 (**c**), and CXCL-2 (**d**) were quantified by ELISA. (**e**,**f**) mRNA levels of NF-κB (**e**) and p38 (**f**) were quantified by qPCR. ^a^ *p* < 0.01, ^b^ *p* < 0.05 versus vehicle group; ^c^ *p* < 0.01, ^d^ *p* < 0.05 versus PM_2.5_ group; ^e^ *p* < 0.01, ^f^ *p* < 0.05 versus PM_2.5_ + DEXA group; TNF-α, tumor necrosis factor α; IL, interleukin; CXCL, C-X-C motif chemokine ligand.

In addition, ELISA using lung homogenate indicated that PM_2.5_ increased the expression of proinflammatory cytokines and chemokines (e.g., TNF-α, IL-6, CXCL-1, and CXCL-2), whereas AsE significantly blocked the induction of proinflammatory mediators by PM_2.5_. The magnitude of decrease in TNF-α and IL-6 by 400 mg/kg AsE was greater than that by DEXA, and there was no difference in all observed proinflammatory mediator levels between PM_2.5_ + DEXA- and PM_2.5_ + AsE (200 mg/kg)-administered group (Figure 6a–d). Moreover, three different doses of AsE significantly blocked PM_2.5_-mediated induction of NF-κB and p38 mRNAs (Figure 6e,f), which are critical signaling molecules for activating inflammation [34,35].

### 3.5. AsE Diminishes Oxidative Stress in the Lungs

**Figure 7:**
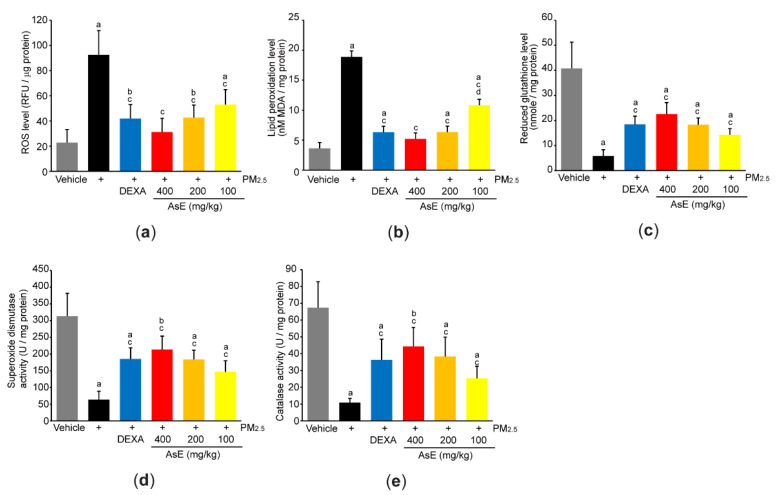
AsE suppresses oxidative stress in the lungs. (**a**) ROS production. Fluorescence intensity was measured after reacting the lung homogenate with 2′,7′-dichlorofluorescein diacetate. (**b**) MDA concentration of the lung homogenate was monitored as an indicator of lipid peroxidation. (**c**–**e**) Antioxidant capacity. Reduced glutathione level (**c**) and superoxide dismutase (**d**) and catalase (**e**) activities were measured from the lung lysates. ^a^ *p* < 0.01, ^b^ *p* < 0.05 versus vehicle group; ^c^ *p* < 0.01 versus PM_2.5_ group; ^d^ *p* < 0.01 versus PM_2.5_ + DEXA group; MDA, malondialdehyde; RFU, relative fluorescence unit; U, unit.

To explore the role of AsE on PM_2.5_-mediated oxidative stress, we first measured ROS production by incubating lung homogenate with 2′,7′-dichlorofluorescein diacetate. As expected, PM_2.5_ significantly increased fluorescence intensity emitted from 2′,7′-dichlorofluorescein, whereas AsE dose-dependently suppressed the ROS production (Figure 7a). Similarly, three different doses of AsE significantly attenuated the increases in lipid peroxidation by PM_2.5_ (Figure 7b). There were no differences in ROS production and lipid peroxidation between the PM_2.5_ + AsE and PM_2.5_ + DEXA groups, except for lipid peroxidation between PM_2.5_ + AsE (100 mg/kg) and PM_2.5_ + DEXA (Figure 7a,b). Moreover, AsE administration significantly prevented PM_2.5_-mediated depletion of antioxidant defense systems such as reduced glutathione, superoxide dismutase, and catalase, and the effect was comparable to that of DEXA (Figure 7c–e).

## 4. Discussion

Although modern scientific evidence related to the pharmacological effects of *A. stricta* has been rarely accumulated, the results of the present study definitely showed that aqueous extract of *A. stricta* root dose-dependently prevented pulmonary congestion, apoptosis, matrix degradation, and sarcomatous changes induced by PM_2.5_ instillation, which provides the evidence that *A. stricta* root is a pulmonary protectant. Various phytochemicals, such as benzoic acids (e.g., vanillic acid 4-β-D-glucopyranoside, syringic acid 4-β-D-glucopyranoside, vanillin, and vanillic acid), coumaric acids (e.g., decursidin), triterpenoids (e.g., methyl adenophorate, lupenone, 24-methylene cycloartenol, sessilifolic acid and sessilifolic acid 3-O-isovalerate), and steroids (e.g., ikusterol and β-sitosterol derivatives) have been identified from the root of *A. stricta* [26,36]. In addition to measuring flavonoid content, we successfully analyzed the level of vanillic acid 4-β-D-glucopyranoside of AsE, and the quantification of vanillic acid 4-β-D-glucopyranoside would be useful to assess the quality of AsE. However, benzoic acid glycosides (e.g., vanillic acid 4-β-D-glucopyranoside) generally have low bioavailability due to the hydrophilicity of the conjugated glycosides. In this regard, it has been believed that the biological effects of ingested glycosylated phytochemicals result from the aglycone metabolites produced during absorption [37,38]. Of various phytochemicals aforementioned, not only vanillic acid, but also syringic acid and vanillin have been reported to protect the lungs from toxic stimuli [39,40,41]. Therefore, aglycosylated benzoic acid derivatives and unidentified phytochemicals (e.g., flavonoids) probably contribute to reducing the pulmonary injuries induced by PM_2.5_, while major bioactive phytochemicals responsible for the pulmonary protection of AsE are needed to be further clarified in the future.

Airway mucus secretion from submucosal glands and secretory epithelial cells is known to be stimulated by acetylcholine and substance P [42,43]. Mucus is an extracellular gel composed primarily of water and mucin. Under normal physiological conditions, mucus contains about 3% of solids, and Muc5AC and Muc5B are the major mucins providing the viscoelasticity of mucus in the airways [31,42]. Mucus is a double-edged sword. As a friend, mucus is the first line of defense to prevent toxins (e.g., PM_2.5_) from penetrating the airway epithelium, and toxic substances entrapped in mucus are expelled from the respiratory system by means of airway ciliary movement and coughing. However, as a foe, highly viscoelastic mucus caused by an excess amount of mucin is difficult to clear from the airways, contributing to the development of all airway diseases [42]. Present results obtained through quantification of mucin and neurotransmitters as well as PAS staining of lung tissue revealed that AsE alleviated PM_2.5_-induced mucus stasis by decreasing mucin synthesis and increasing mucus secretion. To further explore expectorant activity of AsE, phenol red (a pH-sensitive indicator) was intraperitoneally injected into mice 24 h after the last AsE administration, and 250 mg/kg ambroxol hydrochloride, a systematically active mucolytic drug [44,45], was used as a reference. Our supplementary result showed that PM_2.5_ decreased in the redness of body surface, suggesting that PM_2.5_ provokes acidosis through lung injury (Figure S2c). However, PM_2.5_-induced acidosis was significantly prevented by administrating three different doses of AsE (Figure S2c,d). Furthermore, AsE also significantly potentiated the PM_2.5_-dependent phenol red secretion in tracheal lavage fluid (Figure S2e). There was no difference in expectorant activity between ambroxol hydrochloride and AsE (Figure S2c–e). Therefore, mucus clearance is likely the primary explainable mechanism by which AsE protects the lungs via inhibiting the accumulation of inhaled PM_2.5_.

Present results showed that AsE decreased the induction of proinflammatory mediators (e.g., TNF-α, IL-6, CXCL-1, and CXCL-2) by PM_2.5_ in the lungs. In addition, our cytological results using BALF and histological results on hematoxylin- and eosin-stained lung tissues confirmed that AsE can reduce inflammation. TNF-α and IL-6 are acute phase cytokines for amplifying inflammation. In addition, CXCL-1 and CXCL-2 are chemoattracting peptides for the recruitment of polymorphonuclear leukocytes to the injured site [46]. Because NF-κB and p38 mitogen-activated protein kinase are essential signaling molecules for the induction of proinflammatory cytokines as well as the recruitment of inflammatory cells [34,35,47], present results suggest that AsE alleviates lung inflammation through inhibiting NF-κB and p38, albeit detailed cellular pathways and responsible phytochemicals need to be further studied in the future.

In parallel with present results that AsE efficaciously inhibited the ROS production and lipid peroxidation in mice lung receiving PM_2.5_, our supplementary in vitro assay with 2,2-diphenyl-1-picrylhydrazyl (DPPH) revealed that AsE had an ability to directly scavenge radicals generated by DPPH, and IC_50_ of AsE for scavenging DPPH radical was 0.071 ± 0.0075 μg/mL (Figure S3a). In addition, AsE (0.3–3 mg/mL) significantly prevented the decrease in viability of A549 cells, adenocarcinomic cells derived from human alveolar basal epithelium, after exposing the cells to H_2_O_2_ (300 μM) for 12 h, while AsE alone did not affect the viability of A549 cells (Figure S3b). Moreover, significant reduction of ROS production was also proven in A549 cells exposed to H_2_O_2_ in the presence of 1 and 3 mg/mL of AsE (Figure S3c). Especially, AsE treatment in A549 cells not only transactivated the luciferase gene under the control of antioxidant response elements, but also increased the level of heme oxygenase 1 (HO-1) mRNA (Figure S3d), which provides evidence that AsE can activate nuclear factor E2-related factor 2 (Nrf2). Nrf2 has been reported as a master regulator that protects various tissues, including the lung, from oxidative stress [48]. Under the resting state, Nrf2 is present in the cytoplasm and is rapidly degraded through interaction with Kelch-like ECH-associated protein 1 (Keap1). However, electrophiles generated during oxidative stress disrupt the interaction between Nrf2 and Keap1 and allow Nrf2 to translocate into the nucleus, where Nrf2 binds to antioxidant response element and transactivates a battery of genes involved in resisting cells from oxidative stress [48,49]. Antioxidant biomarkers observed in the present study (e.g., reduced glutathione, superoxide dismutase, catalase, and HO-1) have been known to be majorly regulated by Nrf2 [49]. Therefore, it is likely that direct ROS scavenging activity as well as Nrf2-dependent activation of antioxidant defense system in cells cooperatively contribute to the pulmonary protection of AsE against PM_2.5_.

In addition to the role of Nrf2 on antioxidant activity, Nrf2 also participates in orchestrating various biological processes through interplaying with other signaling molecules. Especially, genetic deficiency of Nrf2 has been reported to exacerbate inflammatory responses [50,51,52]. Because NF-κB is also redox-sensitive transcription factor, excessive oxidative stress by Nrf2 knockout amplifies cytokine production through hyperactivation of the NF-κB signaling molecules (e.g., inhibitory κB kinase β) [50,53]. On the contrary, Nrf2 activation has been reported to inhibit NF-κB by competition of transcriptional coactivator complex (e.g., CREB binding protein) and Keap1-mediated sequestration of inhibitory κB kinase β [54,55]. In addition, Nrf2 has been known to regulate the transcription of several cytokine genes via direct binding of Nrf2 to proximal promoter region of these genes [56]. Moreover, decrease in intracellular free iron by Nrf2-mediated HO-1 induction have also been reported to down-regulate NF-κB [57]. Furthermore, Nrf2-dependent p38 inhibition is also involved in the anti-inflammatory mechanism of many natural products [58,59]. Therefore, results from present study suggest that Nrf2 activation by AsE probably contributes to modulating NF-κB and p38 in the lungs.

## 5. Conclusions

The present results demonstrate for the first time the ethnopharmacological relevance of *A. stricta* root as a potent pulmonary protectant by proving that AsE dose-dependently alleviates PM_2.5_-induced pulmonary congestion, alveolar destruction, mucus stasis, inflammation, and oxidative stress. In addition, we compared the pulmonary protective effects of 100–400 mg/kg AsE with that of 0.75 mg/kg DEXA, and the present results indicated that changes for most biomarkers observed in groups receiving 200 or 400 mg/kg of AsE were statistically similar to those by DEXA, with the exception of body weight profile and proinflammatory cytokines. In contrast to DEXA, AsE administration did not change the body weight. Moreover, the magnitude of TNF-α and IL-6 reduction in the mice administered 400 mg/kg of AsE was greater than in the mice administered DEXA. Furthermore, 200 and 400 mg/kg AsE significantly increased acetylcholine and substance P levels in the lungs, which probably contributes to alleviating bronchial mucus retention. Our in vitro supplementary results also suggest that Nrf2 activation by *A. stricta* root may be associated with pulmonary protection from PM_2.5_.

Albeit our results provide evidence that AsE has the potential to protect the lungs in mice exposed to PM_2.5_, the efficacy of AsE in other particulate matters- or toxicants-induced lung disease models, in vivo role of Nrf2 on AsE-dependent pulmonary protection, and the efficacy and safety of AsE in human applications should be further explored to reach more robust conclusion. In addition, although transcriptional control is the predominant mechanism regulating protein expression, protein status and phosphorylation of biomarkers assessed by qPCR in this study need to be further validated. If follow-up studies are successfully carried out, *A. stricta* root would be a promising candidate for preventing or alleviating various respiratory diseases, including lung injury caused by air pollution.

## Figures and Tables

**Table 1:** Primer sequences used in the present study.

Gene Name	Sense Primer	Antisense Primer	RefSeq No.	Amplicon Size (bp)
β-actin	5′-GCTGAGAGGGAAATCG TGCGT-3′	5′-GAAGCATTTGCGGTGCACGATG-3′	NM_007393.5	516
Bcl-2	5′-TGAGAGCAACCGAACGCCCG-3′	5′-CCGTGGCAAAGCGTCCCCTC-3′	NM_009741.5	230
Bax	5′-GGGTGGCAGCTGACATGTTT-3′	5′-GCCTTGAGCACCAGTTTGCT-3′	NM_007527.3	91
Muc5AC	5′-CACCATCTCTACAACCCAAACT-3′	5′-TGAGGTCCAGGTCTTTGTGTCT-3′	NM_010844.3	517
Muc5B	5′-GCCCTCACTGCCTCTGCTCCAC-3′	5′-TTTTACAGTGCCAGGGTTTATT-3′	NM_028801.2	387
NF-κB	5′-CGTTGTTTCCTGGTACAGACC-3′	5′-CCATTTCTTCTTGGTCAAGGG-3′	NM_009045.5	307
p38	5′-CGTTGTTTCCTGGTACAGACC-3′	5′-CCATTTCTTCTTGGTCAAGGG-3′	NM_011951.3	414

NF-κB, nuclear factor κB, Muc5AC, mucin 5AC; Bcl-2, B-cell lymphoma 2; Bax, Bcl-2 associated X.

## Data Availability

Data is contained within the article and Supplementary Materials.

## Supplementary Materials

The following supporting information can be downloaded at: https://www.mdpi.com/article/10.3390/antiox11071376/s1, Figure S1: AsE prevents lung injury caused by PM_2.5_; Figure S2: AsE promotes phlegm excretion; Figure S3: AsE protects A549 cells from oxidative stress.
