# Supplementary material for: Adenophora Stricta Root Extract Protects Lung Injury from Exposure to Particulate Matter 2.5 in Mice
Source: Antioxidants (Basel). 2022 Jul 15;11(7):1376. doi: 10.3390/antiox11071376 (PMC9312037; doi:10.3390/antiox11071376)
Supplement: Supplementary file 1 [file antioxidants-11-01376-s001.zip › antioxidants-1762100-supplementary.pdf]

antioxidants

MDPI

Supplementary information

Adenophora stricta Root Extract Protects Lung Injury from Exposure to Particulate Matter 2.5 in mice

Seok-Man Park [1,2], Cheol-Jong Jung [2], Dae-Geon Lee [1,2], Beom-Rak Choi [3], Tae-Hun Ku [4], Im-Joung La [5], Il-Je Cho [2,*] and Sae-Kwang Ku [1,*]

1 Department of Histology and Anatomy, College of Korean Medicine, Daegu Haany University, Gyeongsan 38610, Korea; smpark@okchundang.co.kr (S.-M.P.); ghost71715@okchundang.co.kr (D.-G.L.)
2 Central Research Center, Okchundang Inc., Daegu 41059, Korea; oc_cjjung@okchundang.co.kr
3 Research Institute, Nutracore Co., Ltd., Gwanggyo SK Viewlake A-3206, Beobjo-Ro 25, Suwon 16514, Korea; brchoi@nutracore.co.kr
4 Okchungdang Korean Medicine Clinic, Ulsan 44900, Korea; oc100002@okchundang.co.kr
5 Atomy R&D Center, Gongju 32511, Korea; imjna@atomyorot.kr
* Correspondence: oc100119@okchundang.co.kr (I.-J.C.); gucci200@dhu.ac.kr (S.-K.K.); Tel.: +82-53-950-0011 (I.-J.C.); +82-53-819-1549 (S.-K.K.)

Supplementary Materials and Methods

Phenol Red Secretion Assay

Experimental procedure for exploring expectorant activity of AsE was additionally approved by the Institutional Animal Care and Use Committee of Daegu Haany University (Approval number, DHU2021-057; Approval date, July 23, 2021). Acclimatization of sixty Balb/c mice, intranasal instillation of $PM_{2.5}$, and oral administration of AsE were conducted identically as already described in the Materials and Methods section, except that 250 mg/kg Ambroxol hydrochloride (AMBR) (Sigma-Aldrich; St. Louise, MO, USA) dissolved in distilled water was used as positive control. The concentration of AMBR was chosen according to the previous report [1]. 10 mL/kg of 5% phenol red (Junsei Chemical Co.; Tokyo, Japan) was intraperitoneally injected at 24 h after the last drug administration. All mice were euthanized by cervical dislocation at 0.5 h after injection of phenol red. An image of entire mouse was captured, and the redness of the mouse skin was measured using an automated image analyzer (*i*Solution FL 9.1, IMT *i*-solution Inc.; Bernaby, BC, Canada). In addition, trachea from the thyroid cartilage to the main stem of bronchi was sonicated for 15 min in 1 mL of saline. 1 mL of 5% $NaHCO_3$ was added to the resulting tracheal lavage fluid, followed by measuring the optical intensity at a wavelength of 546 nm using a Sunrise microplate reader (Tecan; Männedorf, Switzerland).

Histopathology and Immunohistochemistry

General histological process against the left lung lobes (e.g., fixation, trimming, embedding in paraffin, tissue sectioning, hematoxylin and eosin staining, periodic acid–Schiff (PAS) staining), and immunohistochemistry using a cleaved caspase-3 antibody (Cell Signaling Technology; Beverly, MA, USA) were conducted, as previously reported [1,2]. The stained tissue was observed under the Eclipse 80*i* microscope (Nikon, Tokyo, Japan), and mean alveolar surface area (% per mm^2 of lung tissue), thickness of alveolar septum (μm), number of infiltrated inflammatory cells (cells per mm^2 of lung tissue), and number of PAS-positive cells on the secondary bronchus (cells per mm^2 of lung tissue) were counted using an image analyzer (*i*Solution FL 9.1). In addition, the cells showing > 20% of immunoreactivity in

alveolar and secondary bronchus region were counted as the number of cleaved caspase-3 immunoreactive cells (cells per mm^2 of lung tissue).

2,2-Diphenyl-1-picrylhydrazyl (DPPH) Radical Scavenging Assay

Radical scavenging activity of AsE was determined using a DPPH (Sigma-Aldrich, St. Louise, MO, USA), as previously reported [1]. Briefly, 3–300 μg/mL of AsE was incubated with 150 μM DPPH for 0.5 h. Absorbance at a wavelength of 517 nm was measured using a Synergy HTX Multi-Mode Plate Reader (BioTek, Winooski, VT, USA). DPPH radical scavenging activity was calculated as a percentage of the vehicle.

Cell Culture and Viability Assay

A549, a human lung epithelial cell-derived cell, was obtained from American Type Culture Collection (Rockville, MD, USA), and maintained in Dulbecco's modified Eagle's medium (HyClone Laboratories; Logan, UT, USA) containing 10% fetal bovine serum (HyClone Laboratories), 1% Antibiotic-Antimycotic solution (HyClone Laboratories) at 37°C in a humidified atmosphere with 5% CO_2. A549 cells grown in 96-well transparent plate were starved serum for 12 h, and then exposed to 0.3–3 mg/mL AsE for 24 h. To explore cytoprotective activity of AsE, A549 cells pretreated with 0.3–3 mg/mL AsE for 0.5 h were continuously exposed to 300 μM of H_2O_2 for 12 h. After the treated cells were stained with thiazolyl blue tetrazolium bromide (0.1 μg/mL; Sigma-Aldrich) for 4 h, optical intensity of produced formazan crystal was measured at 570 nm of wavelength using a microplate reader (BioTek).

Measurement of ROS Production

The fluorescence intensity was monitored after A549 cells grown in 96-well black plate were treated with AsE (0.3–3 mg/mL), H_2O_2 (300 μM), and 2′,7′-dichlorofluorescein diacetate (10 μM) for 3 h.

Reporter Gene Assay

pGL4.37[luc2P/ARE/Hygro], a reporter plasmid which contains antioxidant response element in the upstream region of the firefly luciferase gene, and pRL-SV40, a *Renilla* luciferase expression plasmid under the control of SV40 promoter, were supplied by Promega (Madison, WI, USA). A549 cells were transiently transfected with 300 ng of pGL4.37[luc2P/ARE/Hygro] and 30 ng of pRL-SV40 for 6 h and exposed to 0.3–3 mg/mL of AsE for 18 h. Luciferase activity in the cell lysates was determined using Dual-Luciferase® Assay System (Promega) and GloMax® 20/20 luminometer (Promega). Relative luciferase activity was calculated by normalizing the luminescence intensity of firefly luciferase to that of *Renilla* luciferase.

qPCR Analysis

Isolation of total RNAs from A549 cells that had been treated with 0.3–3 mg/mL of AsE for 12 h, cDNA synthesis, qPCR, and relative quantification were conducted identically as already described in the Materials and Methods section, except that human specific primer pairs for amplifying heme oxygenase-1 (HO-1) (sense primer, 5′-CAGGAGCTGCTGACCCATGA-3′; antisense, 5′-AGCAACTGTCGCCACCAGAA-3′; RefSeq No. NM_002133.2; amplicon size, 195 bp) and glyceraldehyde 3-phosphate dehydrogenase (GAPDH) (sense, 5′-GAAGGTGAAGGTCGGAGTC-3′; antisense, 5′-GAAGATGGTGATGGGATTTC-3′; RefSeq No. NM_002046.4; amplicon size, 226 bp) were used.

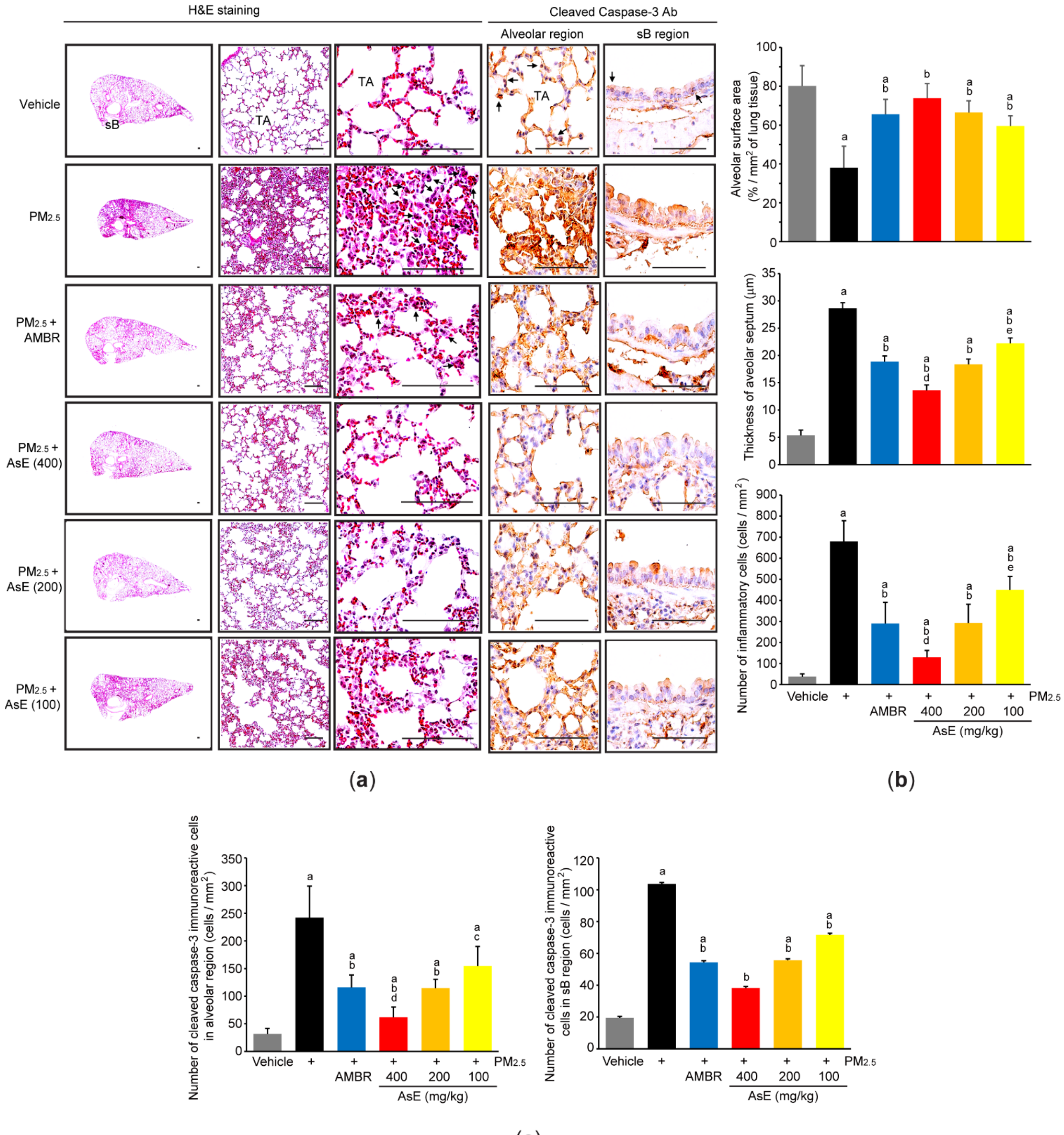

Figure S1. AsE prevents lung injury caused by $PM_{2.5}$. (**a**) Representative histological images. Tissue sections prepared from the left lung lobes were stained using either hematoxylin and eosin (1st to 3rd column) or antibody to cleaved caspase-3 (4th and 5th column). Scale bars indicate 200 μm, and arrows in H&E- and cleaved caspase-3-stained lung tissue indicate $PM_{2.5}$ and immunoreactive cells, respectively. (**b** and **c**) Alveolar surface area (**b**, upper), thickness of alveolar septum (**b**, middle), number of inflammatory cells (**b**, lower), and number of cleaved caspase-3 immunoreactive cells in either alveolar region (**c**, left) or secondary bronchus region (**c**, right) were counted using an image analyzer. $^{a}p < 0.01$ versus vehicle group; $^{b}p < 0.01$, $^{c}p < 0.05$ versus $PM_{2.5}$ group; $^{d}p < 0.01$, $^{e}p < 0.05$ versus $PM_{2.5}$ + AMBR group; Ab, antibody; AMBR, ambroxol hydrochloride; H&E, hematoxylin and eosin; sB, secondary bronchus; TA, terminal respiratory bronchiole-alveoli.

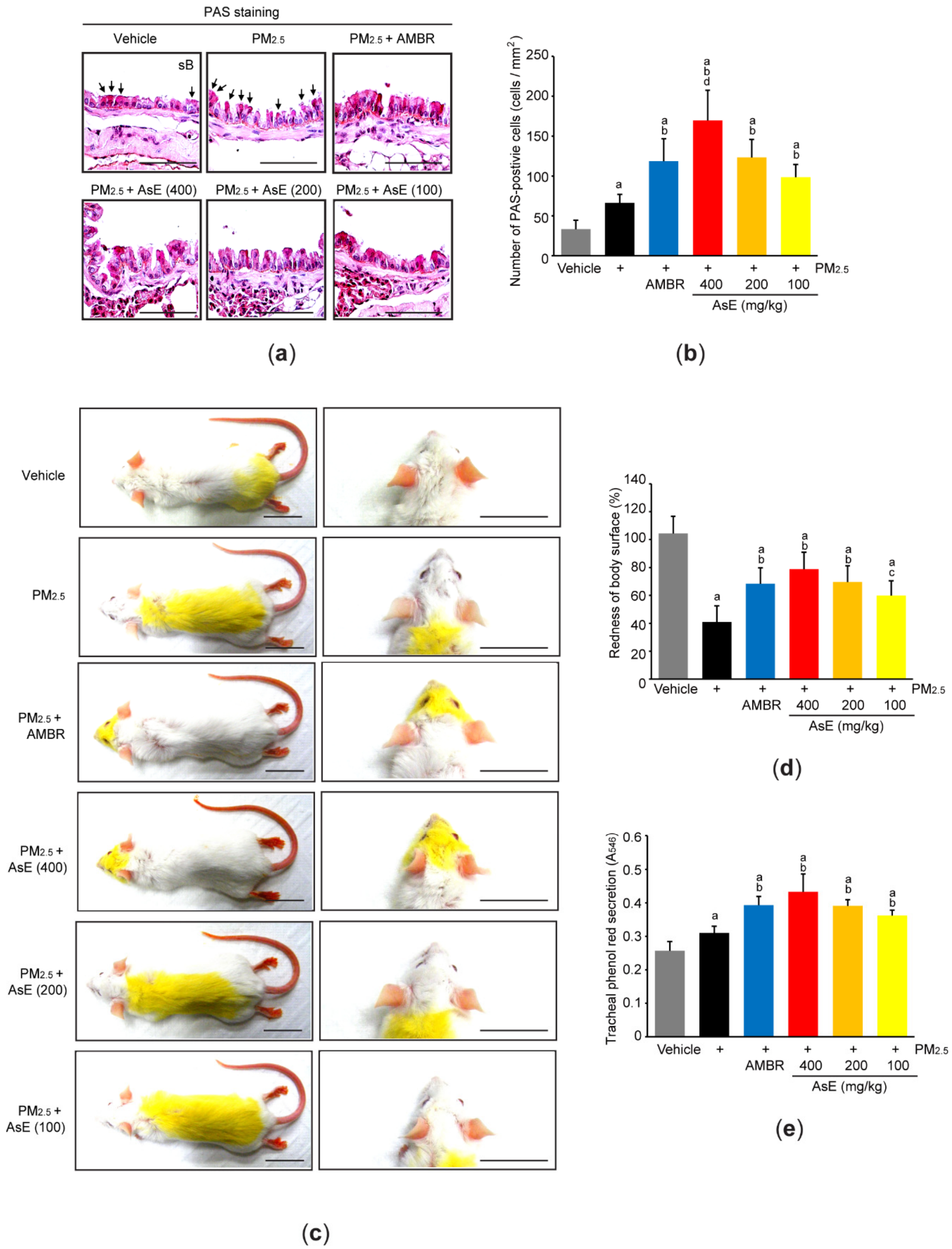

Figure S2. AsE promotes phlegm excretion. (**a**) Representative images of sB region after staining with PAS. Arrows indicate mucus producing cells stained with PAS, and scale bars indicate 200 µm. (**b**) Number of PAS-stained cells was counted using an image analyzer. (**c**–**e**) Phenol red secretion assy. Images of entire mouse (**c**, left) and ear (**c**, right) were captured 30 min after phenol red injection. Scale bars indicate 20 mm. Redness of body surface (**d**) and tracheal lavage fluid (**e**) was measured using image analyzer and spectrophotometer, respectively. [a] $p < 0.01$ versus vehicle group; [b] $p < 0.01$, [c] $p < 0.05$ versus $PM_{2.5}$ group; [d] $p < 0.05$ versus $PM_{2.5}$ + AMBR group; PAS, periodic acid–Schiff.

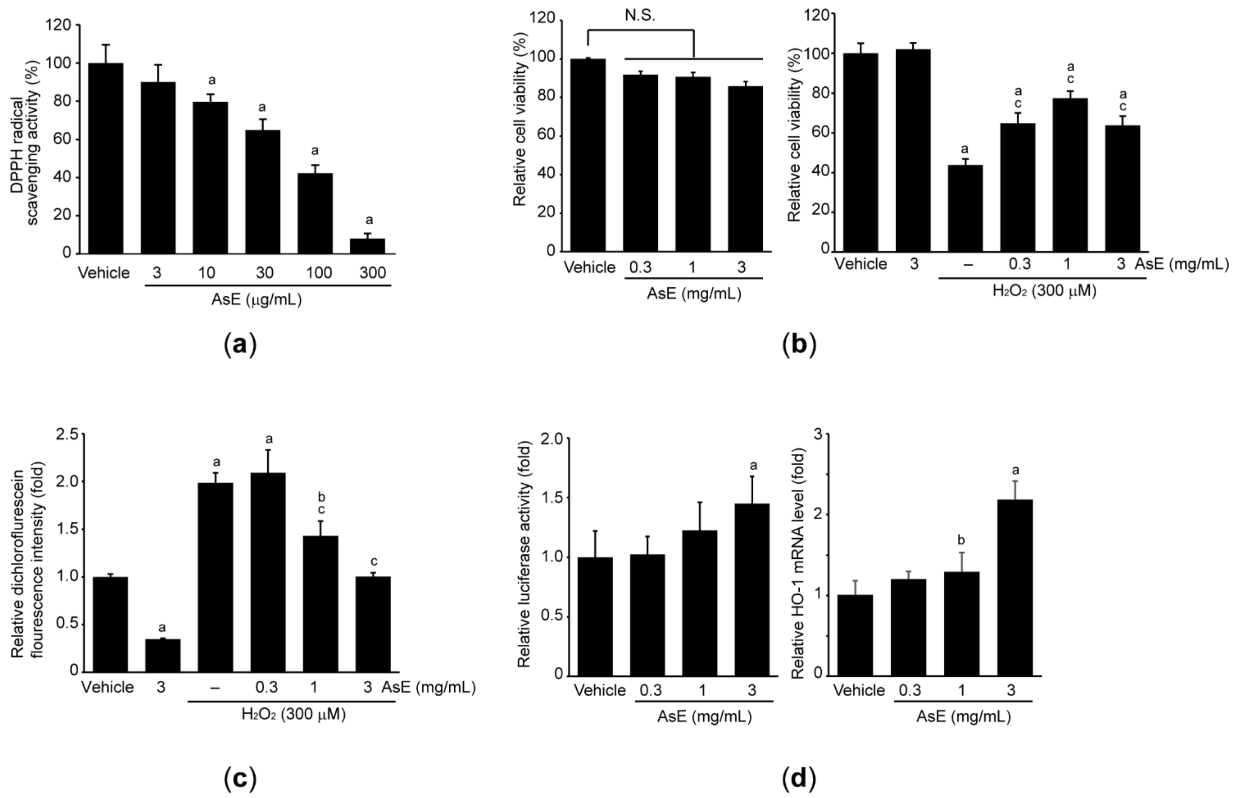

Figure S3. AsE protects A549 cells from oxidative stress. (**a**) The radical scavenging activity was measured after reacting AsE with DPPH. (**b**) MTT assay. A549 cells were incubated with 0.3-3 mg/mL AsE in the presence or absence of 300 μM H_2O_2. (**c**) ROS production. Fluorescence intensity was measured after A549 cells were incubated AsE, H_2O_2, and 2′,7′-dichlorofluorescein diacetate. (**d**) Nrf2 transactivation. A549 cells were transfected with reporter plasmid that contains antioxidant response element, and subsequently exposed to AsE (left). Relative expression of HO-1 was measured after A549 cells were treated with AsE. (right). [a] $p < 0.01$, [b] $p < 0.05$ versus vehicle group; [c] $p < 0.01$ versus H_2O_2; DPPH, 2,2-Diphenyl-1-picrylhydrazyl; HO-1, heme oxygenase 1; MTT, methylthiazole tetrazolium; Nrf2, nuclear factor E2 related factor 2; N.S., not significant.

Supplementary References

1. Hu, J.R.; Jung, C.J.; Ku, S.M.; Jung, D.H.; Bashir, K.M.I. Ku, S.K.; Choi, J.S. Anti-inflammatory, expectorant, and antitussive properties of Kyeongok-go in ICR mice. *Pharm. Biol.* **2021**, *59*, 321–334. https://doi.org/10.1080/13880209.2021.1892155.
2. Cho, I.J.; Shin, J.H.; Choi, B.R.; Park, H.R.; Park, J.E.; Hong, S.H.; Kwon, Y.S.; Oh, W.S.; Ku, S.K. Lemon Balm and Corn Silk Mixture Alleviates Metabolic Disorders Caused by a High-Fat Diet. *Antioxidants* **2022**, *11*, 730. https://doi.org/10.3390/antiox11040730.
